# Brainstem anatomy with 7-T MRI: *in vivo* assessment and *ex vivo* comparison

**DOI:** 10.1186/s41747-023-00389-y

**Published:** 2023-11-16

**Authors:** Graziella Donatelli, Aron Emmi, Mauro Costagli, Paolo Cecchi, Veronica Macchi, Laura Biagi, Marta Lancione, Michela Tosetti, Andrea Porzionato, Raffaele De Caro, Mirco Cosottini

**Affiliations:** 1 Neuroradiology Unit, Azienda Ospedaliero-Universitaria Pisana, Pisa, Italy; 2 Imago7 Research Foundation, Pisa, Italy; 3 Department of Neuroscience, Institute of Human Anatomy, University of Padua, Padua, Italy; 4 Center for Neurodegenerative Disease Research (CESNE), University of Padova, Padua, Italy; 5 Department of Neuroscience, Rehabilitation, Ophthalmology, Genetics, Maternal and Child Sciences (DINOGMI), University of Genoa, Genoa, Italy; 6 Laboratory of Medical Physics and Magnetic Resonance, IRCCS Stella Maris, Pisa, Italy; 7 Department of Translational Research On New Technologies in Medicine and Surgery, Neuroradiology Unit, University of Pisa, 56124 Pisa, Italy

**Keywords:** Brain stem, Grey matter, Magnetic resonance imaging, Staining and labelling, White matter

## Abstract

**Background:**

The brainstem contains grey matter nuclei and white matter tracts to be identified in clinical practice. The small size and the low contrast among them make their *in vivo* visualisation challenging using conventional magnetic resonance imaging (MRI) sequences at high magnetic field strengths. Combining higher spatial resolution, signal- and contrast-to-noise ratio and sensitivity to magnetic susceptibility (*χ*), susceptibility-weighted 7-T imaging could improve the assessment of brainstem anatomy.

**Methods:**

We acquired high-resolution 7-T MRI of the brainstem in a 46-year-old female healthy volunteer (using a three-dimensional multi-echo gradient-recalled-echo sequence; spatial resolution 0.3 × 0.3 × 1.2 mm^3^) and in a brainstem sample from a 48-year-old female body donor that was sectioned and stained. Images were visually assessed; nuclei and tracts were labelled and named according to the official nomenclature.

**Results:**

This *in vivo* imaging revealed structures usually evaluated through light microscopy, such as the accessory olivary nuclei, oculomotor nucleus and the medial longitudinal fasciculus. Some fibre tracts, such as the medial lemniscus, were visible for most of their course. Overall, in *in vivo* acquisitions, χ and frequency maps performed better than T2*-weighted imaging and allowed for the evaluation of a greater number of anatomical structures. All the structures identified *in vivo* were confirmed by the *ex vivo* imaging and histology.

**Conclusions:**

The use of multi-echo GRE sequences at 7 T allowed the visualisation of brainstem structures that are not visible in detail at conventional magnetic field and opens new perspectives in the diagnostic and therapeutical approach to brain disorders.

**Relevance statement:**

*In vivo* MR imaging at UHF provides detailed anatomy of CNS substructures comparable to that obtained with histology. Anatomical details are fundamentals for diagnostic purposes but also to plan a direct targeting for a minimally invasive brain stimulation or ablation.

**Key points:**

• The *in vivo* brainstem anatomy was explored with ultrahigh field MRI (7 T).

*• In vivo* T2*-weighted magnitude, χ, and frequency images revealed many brainstem structures.

*• Ex vivo* imaging and histology confirmed all the structures identified *in vivo*.

• χ and frequency imaging revealed more brainstem structures than magnitude imaging.

**Graphical Abstract:**

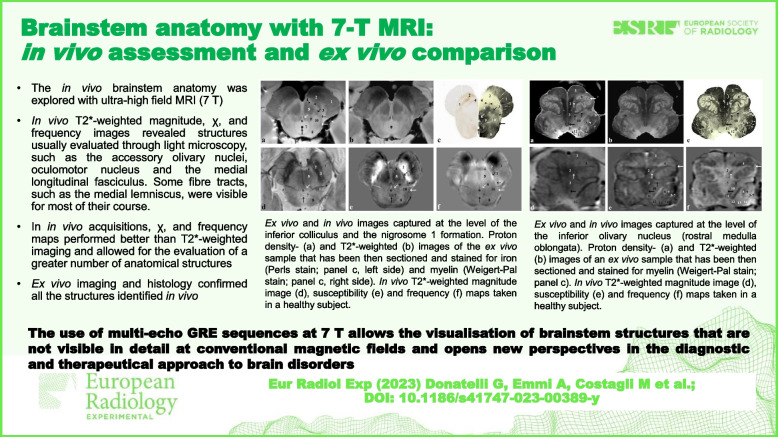

**Supplementary Information:**

The online version contains supplementary material available at 10.1186/s41747-023-00389-y.

## Background

The brainstem is an anatomical structure containing several functional systems and a myriad of nuclei and connecting fibre bundles involved in many different functional pathways spanning from voluntary movement to epicritic and protopathic sensibility, from respiratory and cardiovascular control to the regulation of wakefulness and consciousness [1]. The precise identification of the substructures constituting each anatomo-functional system would be extremely useful for diagnostic and therapeutical purposes. Lesions affecting the brainstem (such as inflammatory plaques, vascular lacunes, tumours, or infections) can selectively damage anatomical pathways and cause devastating consequences even if they are small. Moreover, an accurate identification of the neuromodulatory centres in the brainstem is fundamental to plan a direct targeting approach for brain stimulation and to monitor the response to invasive [2] and noninvasive stimulation and to pharmacological therapies [1, 3, 4].

So far, in the clinical practice, the localisation of brainstem substructures is inferred based on histological atlases [5] and their presumed proximity to surface landmarks. Conventional magnetic resonance imaging (MRI) at clinical magnetic fields depicts the brainstem as a homogenous structure where grey and white matter components are not distinguishable from each other. The uniform aspect of the brainstem is related to the small dimensions of the complex substructures which lie there and the low contrast among them. MRI with unconventional contrasts and higher spatial resolution, instead, could improve the detection of both grey and white matter structures.

The *in vivo* MRI localisation of white matter fibre bundles within the brainstem can be obtained with diffusion tensor imaging [6], but the contemporary visualisation of both white matter tracts and grey matter nuclei is difficult. For this purpose, track density imaging has been combined with proton density (PD) and T2 mapping, but, notwithstanding the increased contrast, the location of several nuclei has remained presumed and their identification based on the topography of fibre bundles or anatomical surface markers [7]. Recently, a customised three-dimensional (3D) fast grey matter acquisition T1 inversion recovery for myelin suppression has provided good quality images with a 3-T scanner. The isotropic voxel allowed optimal multiplanar reformatting, but the spatial resolution was in the order of the millimetre (0.8 mm) [8].

A promising technique to explore the brain anatomy *in vivo* is the multi-echo gradient-recalled-echo (GRE) sequence, which provides an impressive image contrast resulting primarily from iron and myelin content of the tissue [9]. In this sequence, T2*-weighted magnitude, filtered phase information, or both [10] are used to produce images sensitive to magnetic susceptibility (χ). Besides, *in vivo* quantitative χ maps obtained from the phase of the MRI signal [11] can be used to explore brain and brainstem anatomy [12]. The introduction of ultrahigh field (7-T) MRI can further improve the ability to detect the brainstem substructures *in vivo* using susceptibility-weighted imaging, as sensitivity to susceptibility phenomena and spatial resolution of images increase with increasing static magnetic field strength [13].

Here, we explored the brainstem anatomy in a healthy volunteer using T2*-weighted magnitude, χ, and frequency maps obtained with a multi-echo GRE sequence at 7 T. Then, anatomical details of grey matter nuclei and white matter fibre bundles were compared with those captured in PD- and T2*-weighted images of an *ex vivo* sample and finally confirmed with the examination of stained histological sections.

## Methods

We acquired high-resolution 7-T MR images of the brainstem in a healthy volunteer, and, to compare *in vivo* images with a standard of reference, we built an anatomical ground truth by acquiring high-resolution images of an isolated brainstem sample deriving from the Body Donation Program of the Institute of Human Anatomy of the University of Padova [14], which was subsequently sectioned for histological staining. The identification of anatomical structures and substructures on *ex vivo* images was considered correct if confirmed by microscopic examination of the sectioned sample.

The study was approved by the local ethics committees (Padua: Body Donation Program Ethical Standards; CEAVNO: protocol number 17664), and the volunteer gave the written informed consent to the enrolment. The *ex vivo* specimen was handled according to the ethical standards and regulations of the Body Donation Program of the University of Padova and in accordance to the Declaration of Helsinki and the Italian Law 10/2020 on Body Donation [15, 16].

### *In vivo* acquisition and data processing

A 46-year-old healthy female volunteer underwent brain MRI with a 7-T MR950 system (General Electric Healthcare, Milwaukee, WI, USA) equipped with a 2ch-tx/32ch-rx head coil (Nova Medical, Wilmington, MA, USA).

The protocol included a 3D multi-echo GRE sequence targeting the brainstem and prescribed perpendicularly to the floor of the fourth ventricle, with the following technical parameters: time of repetition (TR) 54.1 ms; times of echo (TEs) 7.3 ms, 15.5 ms, 23.6 ms, 31.8 ms, and 40.0 ms, flip angle 15° and “array coil spatial sensitivity encoding” parallel imaging acceleration 2; spatial resolution 0.3 × 0.3 × 1.2 mm^3^; number of excitations (NEX) 0.7; and scan duration 12′ 11″.

The complex-valued data obtained at each TE were used to produce images with T2*-weighted contrast, phase information and a χ map, following an established procedure previously described [17]. In brief, the real and imaginary parts of the images were converted into magnitude and phase; the average of the magnitude images obtained at each TE provided 3D images with T2*-weighted contrast. The phase images obtained at each TE underwent Laplacian-based phase unwrapping [18, 19] and background phase removal via variable-size spherical-kernel harmonic artifact reduction (V-SHARP) [20, 21]; after normalisation by the TE of each echo, they were averaged to obtain one resultant frequency image. The quantitative χ map was computed by averaging the χ images obtained by applying the iLSQR method [18] to the unwrapped and V-SHARPed phase data of each individual echo [22].

### *Ex vivo* acquisition

One human brain from a 48-year-old body donor was sampled after a 48-h post-mortem delay and fixed in 4% paraformaldehyde for 30 days. The brain was placed in an airtight cylinder filled with perfluoropolyether, and air bubbles and paraformaldehyde residual drops were accurately removed. After imaging the midbrain, the brainstem was anatomically sectioned at the level of the ponto-mesencephalic junction, placed in a smaller cylinder filled with perfluoropolyether and imaged using targeted sequences.

The *ex vivo* MRI acquisitions were performed with the same scanner described before. The MRI protocol included high-resolution sequences for morphologic and anatomical purposes and images tailored to create sample-specific cutting boxes.

The midbrain was imaged using the 2ch-tx/32ch-rx head coil. The high-resolution sequences included whole brain two-dimensional (2D) spin-echo (SE) PD-weighted images (TR 1,500 ms; TE 21 ms; in-plane resolution 0.33 × 0.33 mm^2^; slice thickness 1.2 mm; NEX 2; overall scan duration for two interleaved series = 1 h 43′ 36″), and a 3D multi-echo GRE sequence (TR 54.1 ms; TEs 8.9 ms, 19.7 ms, and 30.5 ms; spatial resolution 0.3 × 0.3 × 0.3 mm^3^, NEX 0.7; scan duration 1 h 23′ 55″). An additional 2D SE PD-weighted acquisition (TR 1500 ms; TE 21 ms; in-plane resolution 0.39 × 0.39 mm^2^: slice thickness 1.2 mm; NEX 1; scan duration 40′ 30″) was performed and used to design and create a tailor-made cutting box [23] for the midbrain.

Pons and medulla oblongata were imaged using a custom-built Tx/Rx birdcage coil with the bore diameter of 48 mm [24]. As for the midbrain, the acquisition protocol included 2D SE PD-weighted images (TR 1,500 ms; TE 21 ms; in-plane resolution 0.16 × 0.16 mm^2^; slice thickness 1 mm; NEX 4; overall scan duration for two interleaved series 1 h 42′ 36″) and a 3D multi-echo GRE sequence (TR 59.2 ms; TEs 9.5 ms, 19.8 ms, and 30.2 ms; spatial resolution 0.2 × 0.2 × 0.2 mm^3^; NEX 0.7; scan duration 52′). Another 2D SE PD-weighted acquisition (TR 1500 ms; TE 21 ms; in-plane resolution 0.16 × 0.16 mm^2^; slice thickness 1 mm; NEX 1, scan duration 27′) was performed and used to design and print a cutting box [23] dedicated to medulla oblongata and pons.

All the 2D sequences were prescribed perpendicularly to the floor of the fourth ventricle.

### Histological staining

The cutting boxes were designed to hold the samples during anatomical sectioning, allowing the perfect match between each transverse section and specific MRI images. The floor of the cutting boxes perfectly fitted the corresponding surface of the samples, while the walls had fissures at fixed steps that were used as guides for the sectioning. The anatomical levels of cutting planes were selected based on imaging in order to obtain 6–10-mm-thick macrosections and avoid splitting the main structures of interest into two parts. The specimen was then sectioned; embedded in paraffin; stained with haematoxylin and eosin, Klüver-Barrera, Weigert-Pal and Perls; and used as reference for MRI analysis.

### Image analysis

Six axial anatomical levels were selected for image analysis, the same in each *in vivo* dataset (T2*-weighted magnitude images, χ, and frequency maps), in the *ex vivo* images and in the corresponding neuroanatomical slices. They were as follows: the rostral midbrain, at the level of the red nucleus; the caudal midbrain, at the level of the inferior colliculus and the nigrosome 1 formation; the rostral pons, where the superior cerebellar peduncles finish to enter the brainstem; the caudal pons, at the level of the abducens nucleus; the rostral medulla oblongata, at the level of the mid-portion of the inferior olivary nucleus; and the caudal medulla oblongata, just below the inferior olivary nucleus.

*Ex vivo* and *in vivo* images were assessed by two neuroradiologists with 13 and 26 years of experience, each dataset in a separate session; nuclei and tracts identified by consensus were labelled and named according to the official nomenclature [25]. In the meantime, an experienced neuroanatomist analysed histological staining of the selected *ex vivo* slices to confirm nomenclature and position of the brainstem substructures.

## Results

**Fig. 1:**
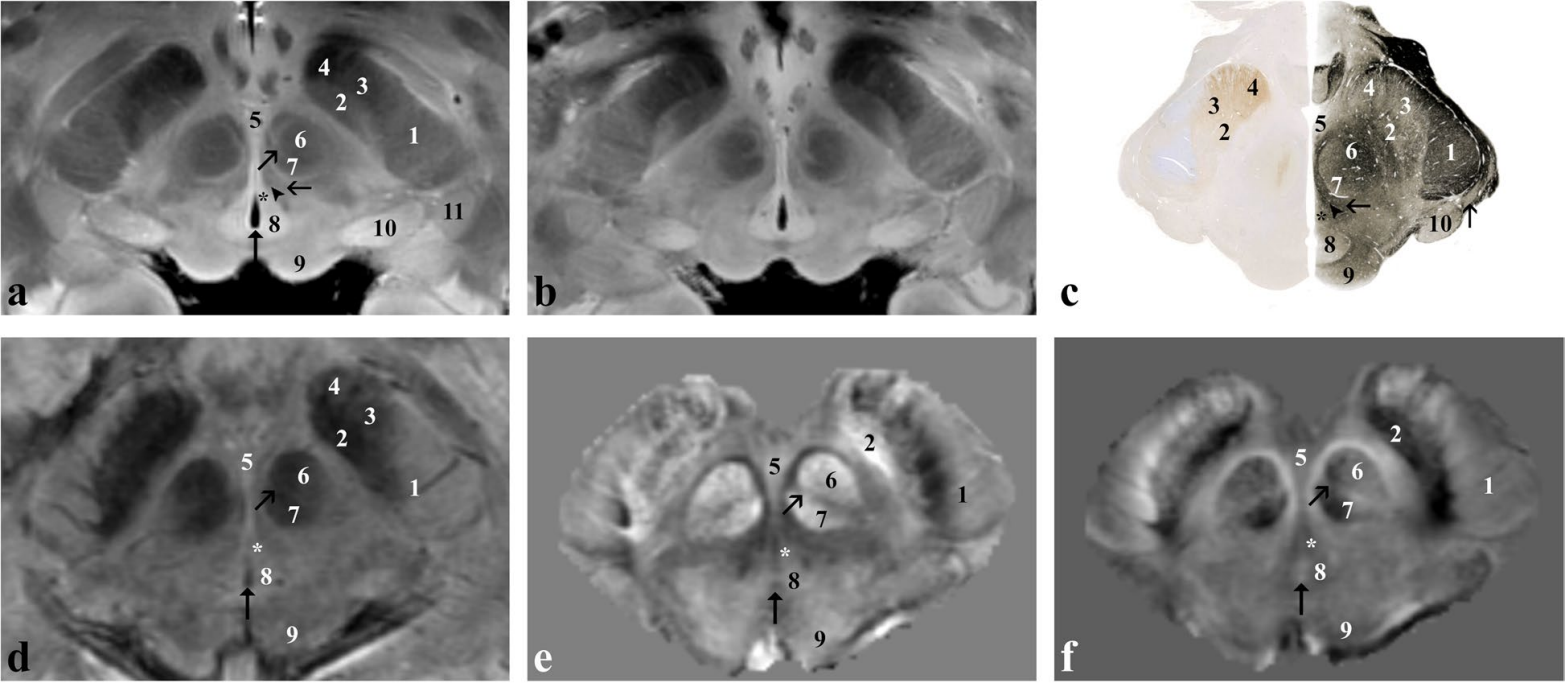
*Ex vivo* and *in vivo* images captured at the level of the red nucleus (rostral midbrain). Proton density**-** (**a**) and T2*-weighted (**b**) images of the *ex vivo* sample that has been then sectioned and stained for iron (Perls stain; **c** left side) and myelin (Weigert-Pal stain; **c** right side). *In vivo* T2*-weighted magnitude image (**d**), susceptibility (**e**) and frequency (**f**) maps taken in a healthy subject. 1, corticospinal, corticonuclear, frontopontine and parietotemporopontine tracts; 2, subthalamic nucleus; 3, pars reticulata of the substantia nigra; 4, extension of the pars reticulata into the crus cerebri; 5, ventral tegmental area; 6, magnocellular portion of the red nucleus; 7, parvocellular portion of the red nucleus; 8, periaqueductal grey matter; 9, superior colliculus; 10, medial geniculate body; 11 (vertical open black arrow in **c**), lateral geniculate body; oblique open black arrow, lamina medullaris of the red nucleus; arrowhead, medial longitudinal fasciculus; horizontal open black arrow, central tegmental tract; vertical black arrow, cerebral aqueduct; asterisk, oculomotor, principal oculomotor and accessory oculomotor nuclei

**Fig. 2:**
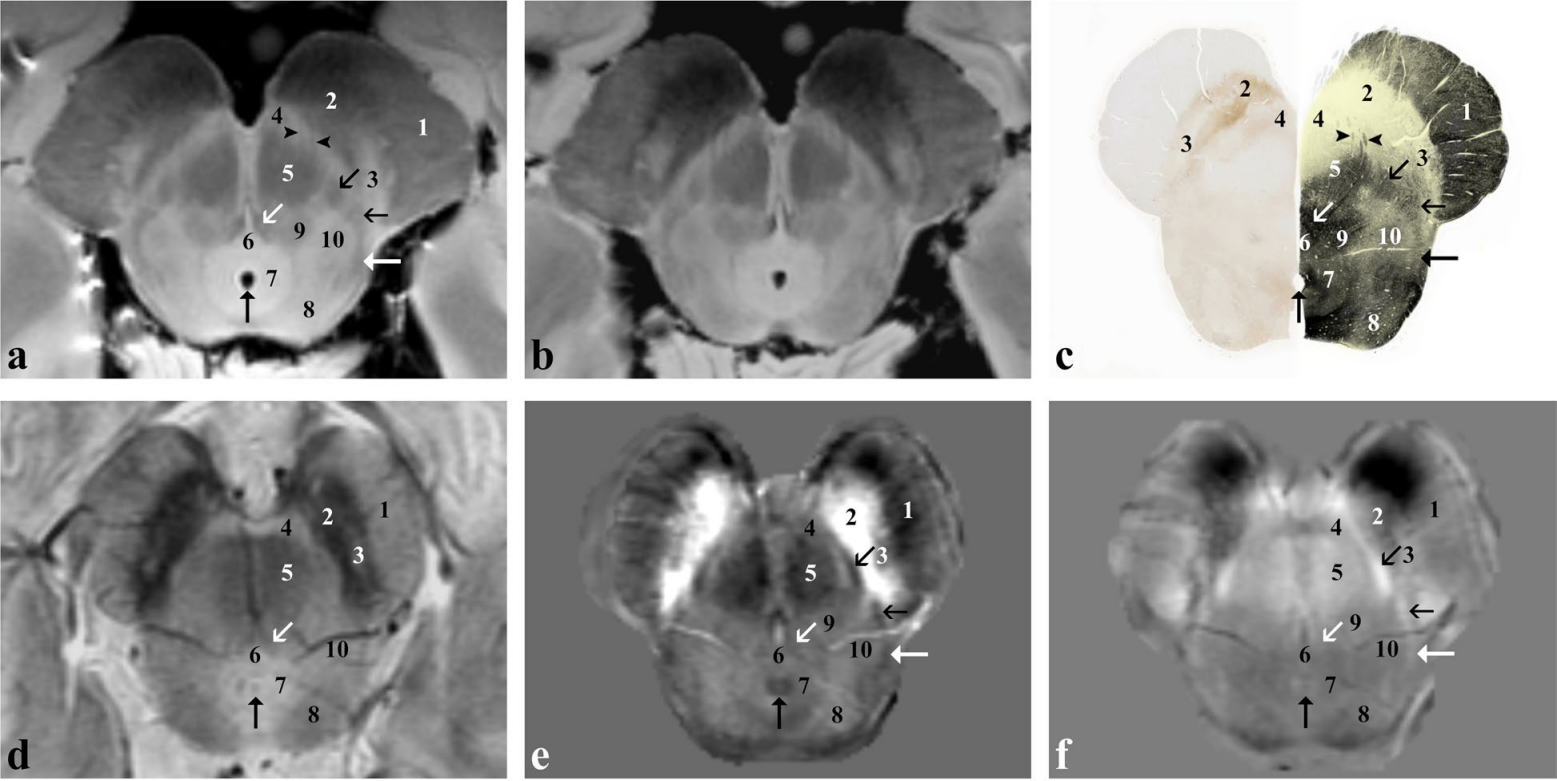
*Ex vivo* and *in vivo* images captured at the level of the inferior colliculus and the nigrosome 1 formation (caudal midbrain). Proton density- (**a**) and T2*-weighted (**b**) images of the *ex vivo* sample that has been then sectioned and stained for iron (Perls stain; **c** left side) and myelin (Weigert-Pal stain; **c** right side). *In vivo* T2*-weighted magnitude image (**d**), susceptibility (**e**) and frequency (**f**) maps taken in a healthy subject. 1, corticospinal, corticonuclear, frontopontine and parietotemporopontine tracts; 2, substantia nigra; 3, nigrosome 1; 4, ventral tegmental area; 5, superior cerebellar peduncle (or brachium conjunctivum); 6, dorsal nucleus of raphe; 7, periaqueductal grey matter; 8, inferior colliculus; 9, central tegmental tract; 10, nucleus reticularis cuneiformis and nucleus reticularis peduncolopontinus; arrowheads, fibres of the oculomotor nerve; oblique open black arrow, medial lemniscus; horizontal open black arrow, spinothalamic tract; horizontal white arrow (horizontal black arrow in **c**), lateral lemniscus; oblique open white arrow, tectospinal tract and medial longitudinal fasciculus; vertical black arrow, cerebral aqueduct

**Fig. 3:**
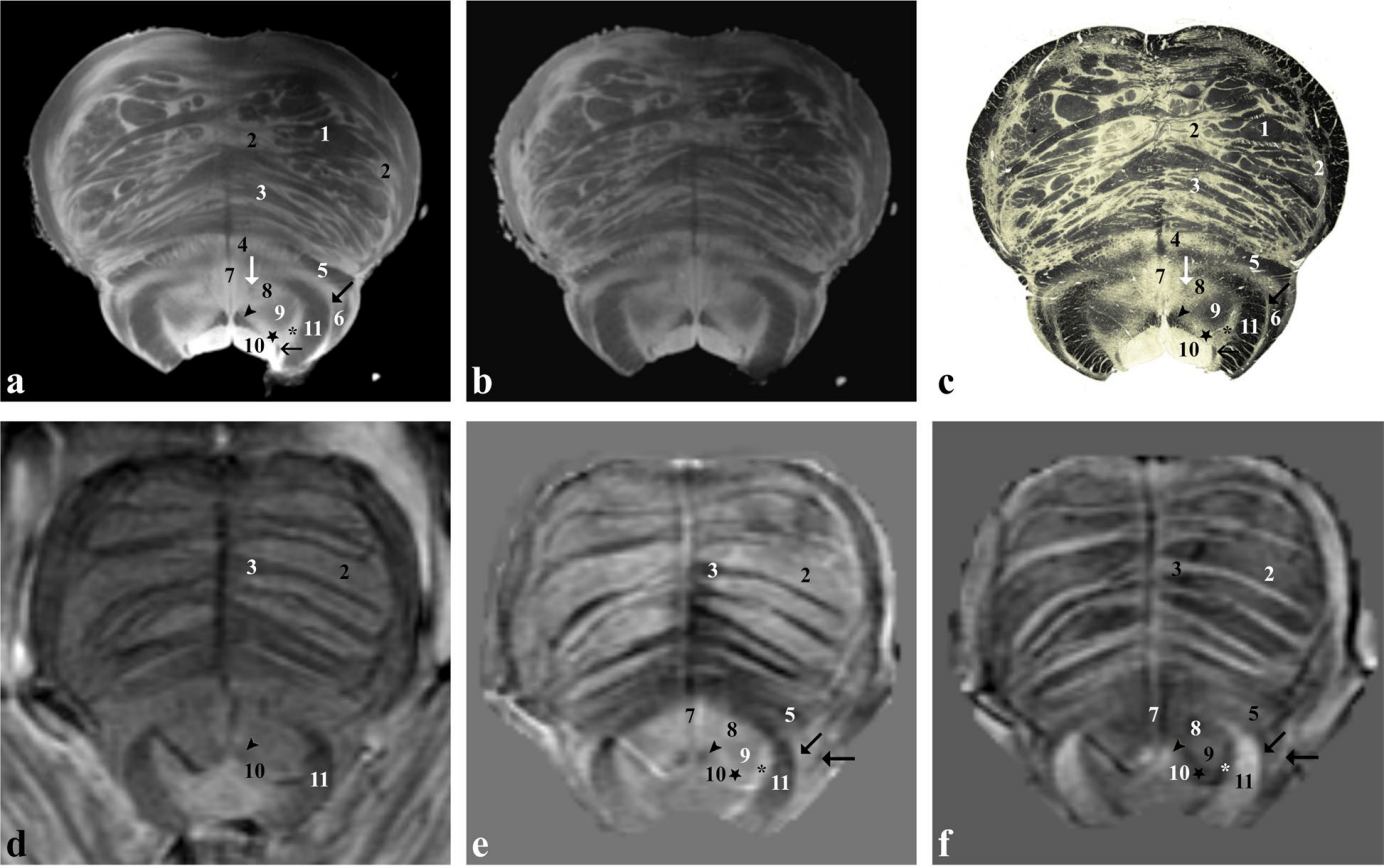
*Ex vivo* and *in vivo* images captured at the level of the superior cerebellar peduncles (rostral pons). Proton density- (**a**) and T2*-weighted (**b**) images of the *ex vivo* sample that has been then sectioned and stained for myelin (Weigert-Pal stain; **c**). *In vivo* T2*-weighted magnitude image (**d**), susceptibility (**e**) and frequency (**f**) maps taken in a healthy subject. 1, corticospinal tract split into small fasciculi, corticonuclear, frontopontine and parietotemporopontine tracts; 2, pontine nuclei; 3, pontocerebellar fibres; 4, nucleus reticularis tegmenti pontis; 5, medial lemniscus and spinothalamic tract; 6, lateral lemniscus (horizontal black arrow in **e** and **f**); 7, nucleus reticularis centralis superior; 8, nucleus reticularis pontis oralis; 9, central tegmental tract; 10, central grey matter; 11, superior cerebellar peduncle (or brachium conjunctivum); white arrow, tectospinal tract; black arrowhead, medial longitudinal fasciculus; open black arrow, mesencephalic trigeminal tract; oblique black arrow, lateral parabrachial nucleus; star, nucleus coeruleus; asterisk, medial parabrachial nucleus and nucleus subcoeruleus

**Fig. 4:**
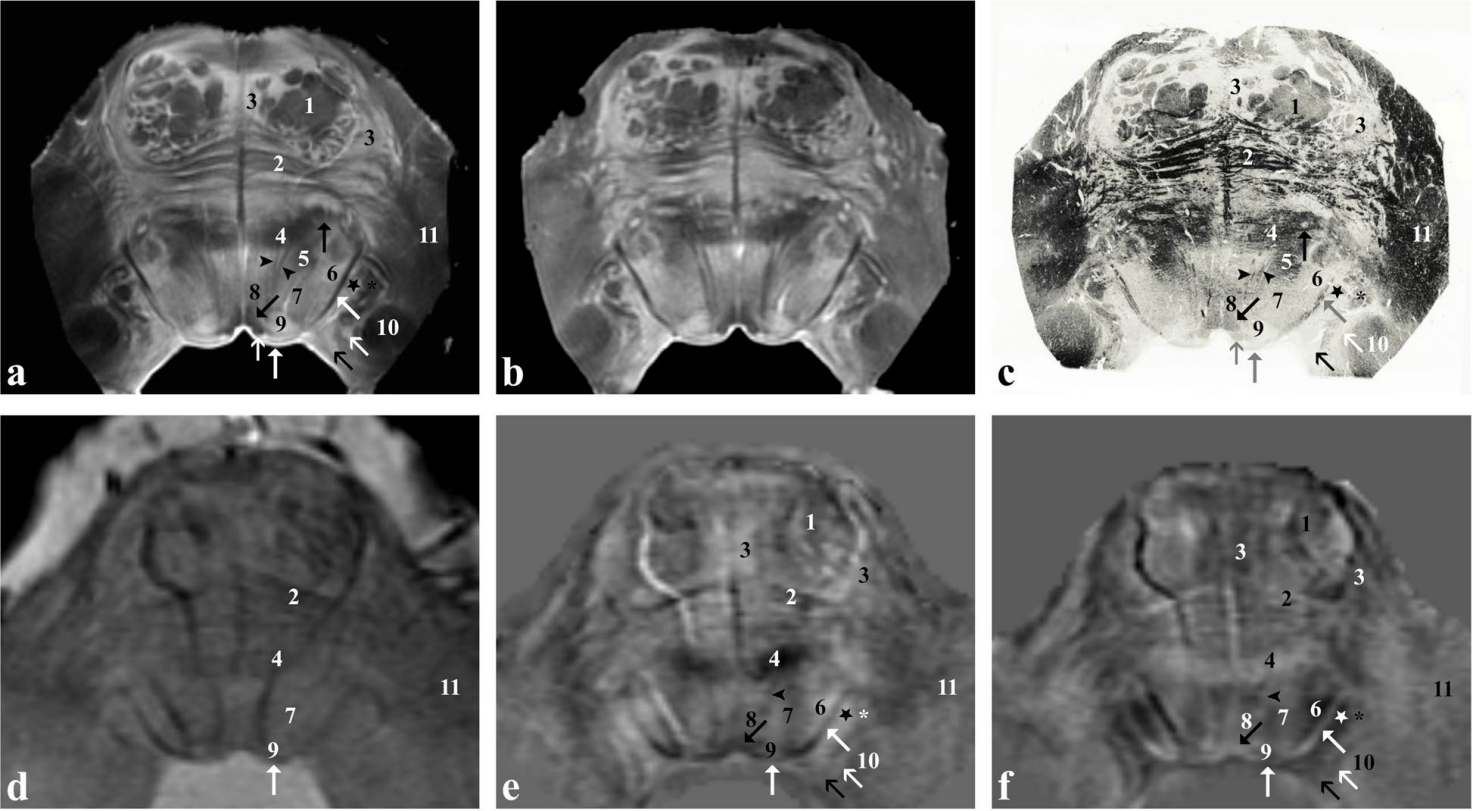
*Ex vivo* and *in vivo* images captured at the level of the abducens nucleus (caudal pons). Proton density- (**a**) and T2*-weighted (**b**) images of an *ex vivo* sample that has been then sectioned and stained for myelin (Weigert-Pal stain; **c**). *In vivo* T2*-weighted magnitude image (**d**), susceptibility (**e**) and frequency (**f**) maps taken in a healthy subject. 1, corticospinal tract split into small fasciculi and corticonuclear tract; 2, pontocerebellar fibres; 3, pontine nuclei; 4, medial lemniscus; 5, central tegmental tract; 6, facial nucleus; 7, pontine reticularis nuclei; 8, tectospinal tract; 9, abducens nucleus; 10, inferior cerebellar peduncle (or corpus restiform); 11, middle cerebellar peduncle; vertical black arrow, spinothalamic tract and lateral lemniscus; oblique black arrow, medial longitudinal fasciculus; vertical white arrow (vertical grey arrow in **c**), facial colliculus; oblique white arrow (oblique grey arrow in **c**), fibres of the facial nerve; vertical open white arrow (vertical open grey arrow in **c**), dorsal longitudinal fasciculus; oblique open white arrow, lateral vestibular nucleus; oblique open black arrow, medial and superior vestibular nuclei; arrowheads, fibres of the abducens nerve; star, spinal trigeminal nucleus; asterisk, spinal trigeminal tract

**Fig. 5:**
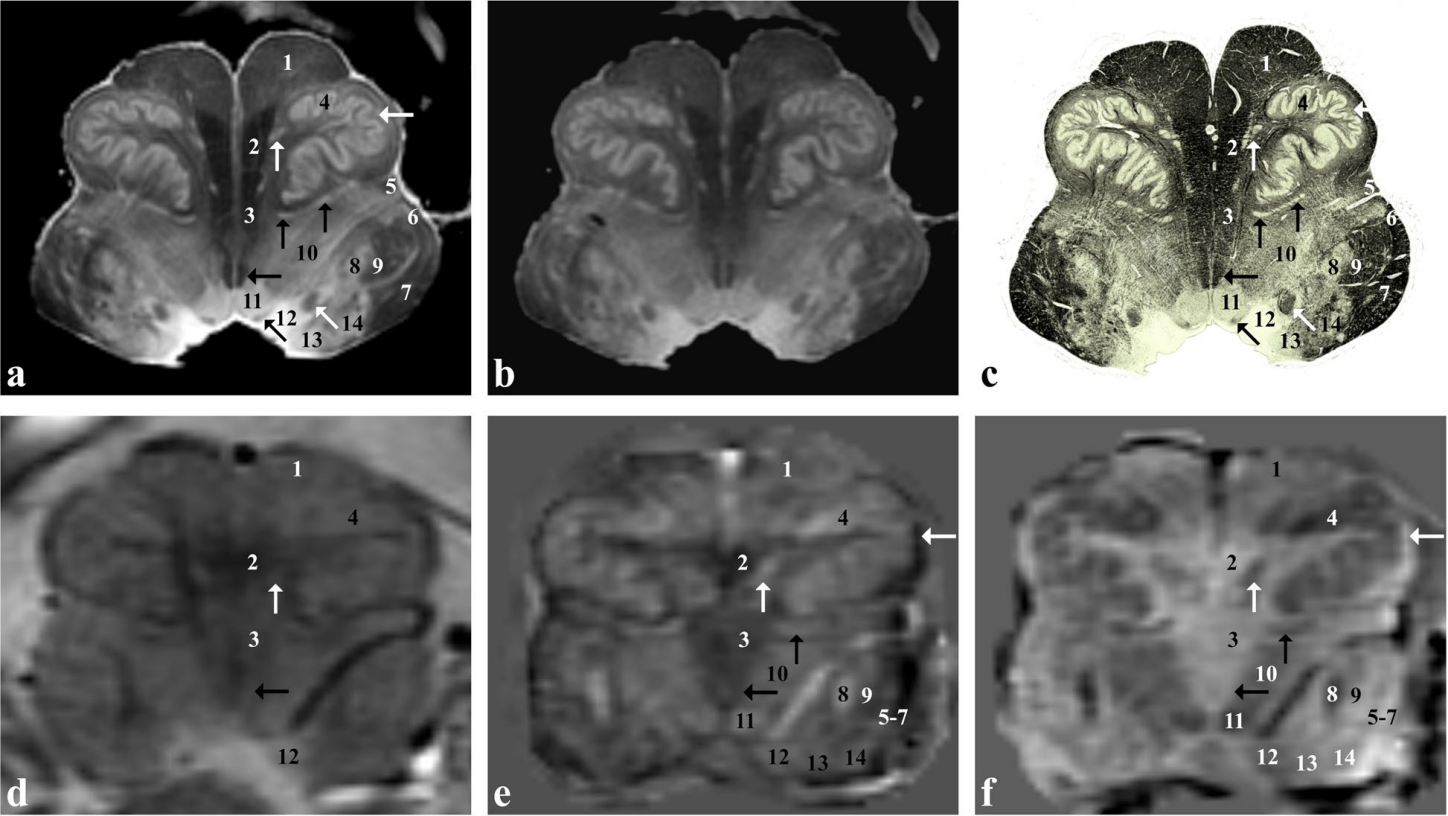
*Ex vivo* and *in vivo* images captured at the level of the inferior olivary nucleus (rostral medulla oblongata). Proton density- (**a**) and T2*-weighted (**b**) images of an *ex vivo* sample that has been then sectioned and stained for myelin (Weigert-Pal stain; **c**). *In vivo* T2*-weighted magnitude image (**d**), susceptibility (**e**) and frequency (**f**) maps taken in a healthy subject. 1, corticospinal tract; 2, medial lemniscus; 3, tectospinal tract; 4, inferior olivary nucleus; 5, spinothalamic tract; 6, ventral spinocerebellar tract; 7, inferior cerebellar peduncle (or corpus restiform); 8, spinal trigeminal nucleus; 9, spinal trigeminal tract; 10, nucleus reticularis centralis medullae oblongatae; 11, hypoglossal nucleus; 12, dorsal motor nucleus of the vagus and nucleus of the solitary tract; 13, medial vestibular nucleus; 14, lateral cuneate nucleus; vertical white arrow, medial accessory olivary nucleus; horizontal white arrow, amiculum of the inferior olivary nucleus; vertical black arrow, dorsal accessory olivary nucleus; horizontal black arrow, medial longitudinal fasciculus; oblique black arrow, dorsal longitudinal fasciculus; oblique white arrow, solitary tract

**Fig. 6:**
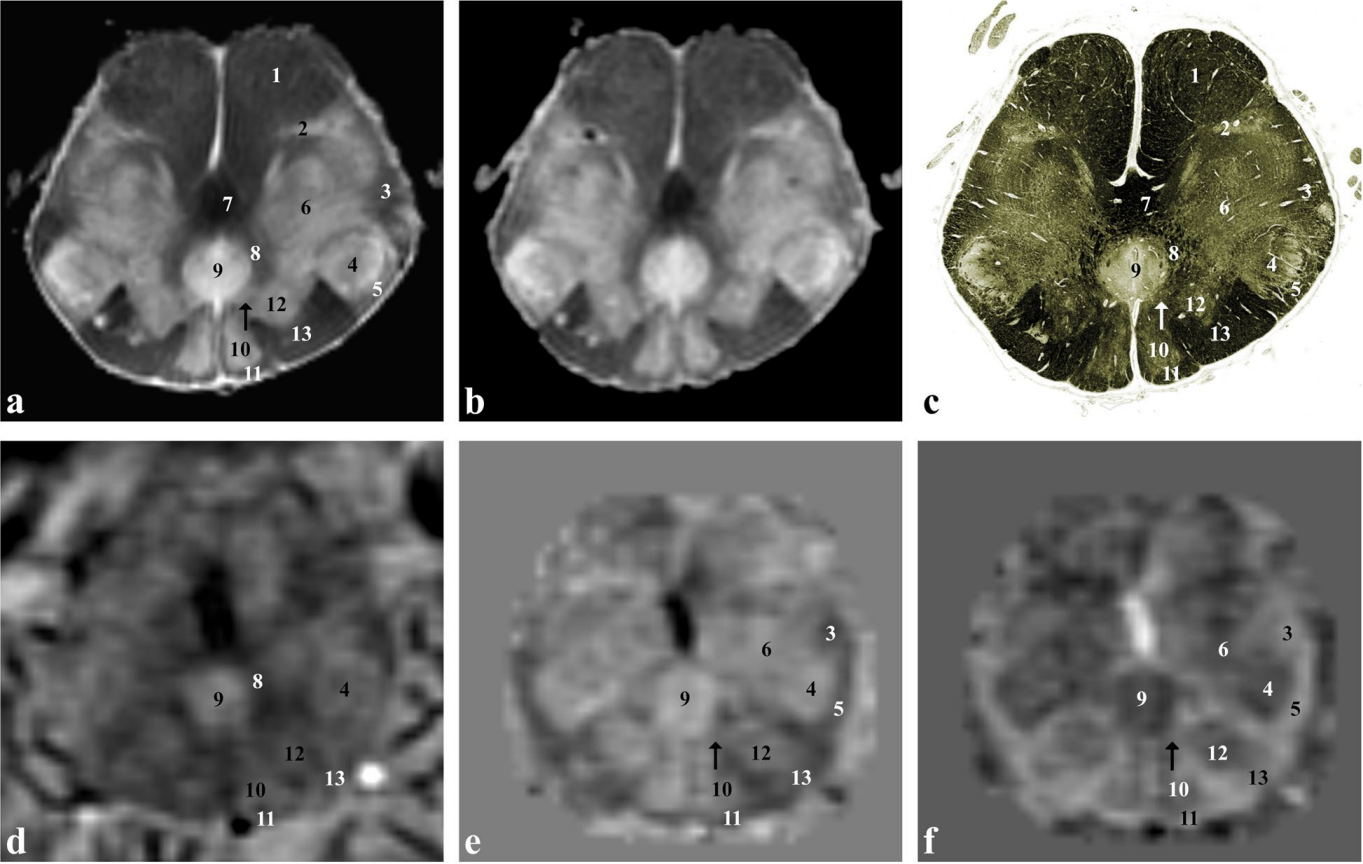
*Ex vivo* and *in vivo* images captured just below the inferior olivary nucleus (caudal medulla oblongata). Proton density- (**a**) and T2*-weighted (**b**) images of an *ex vivo* sample that has been then sectioned and stained for myelin (Weigert-Pal stain; **c**). *In vivo* T2*-weighted magnitude image (**d**), susceptibility (**e**) and frequency (**f**) maps taken in a healthy subject. 1, corticospinal tract; 2, medial accessory olivary nucleus (caudal part); 3, spinothalamic tract, ventral spinocerebellar tract, dorsal spinocerebellar tract and rubrospinal tract; 4, spinal trigeminal nucleus; 5, spinal trigeminal tract; 6, nucleus reticularis centralis medullae oblongatae; 7, medial lemniscus; 8, internal arcuate fibres; 9, central canal; 10, gracile nucleus; 11, gracile fasciculus; 12, medial cuneate nucleus; 13, cuneate fasciculus; black arrow (white arrow in **c**), nucleus of the solitary tract

Several grey matter nuclei and white matter fibre tracts of the brainstem were visible in *in vivo* 7-T MR images. They are summarised in Supplementary Table S1, labelled in Figs. 1, 2, 3, 4, 5 and 6 and described in the text together with the anatomo-functional systems of which they are part.

At the rostral midbrain level (Fig. 1), the red nucleus and the subthalamic nucleus stand out because of their high iron content. Within the red nucleus, the magnocellular (6) and parvocellular (7) portions are distinguishable from each other in both *ex vivo* and *in vivo* images because of the higher iron content compared to that of the interposed lamina medullaris (oblique open black arrow). The lens-shaped subthalamic nucleus (2) is visible at the medial edge of the cerebral peduncle (1). The latter is formed by the frontopontine tract (medial 1/5), the pyramidal pathway (central 3/5) and the parieto-temporo-occipito-pontine tract (lateral 1/5). At the level of the tegmentum, the prerubral field and the white matter surrounding the red nucleus, belonging mainly to the superior cerebellar peduncle, the rubro-spinal and the rubro-thalamocortical pathways can be appreciated. Posteriorly to the red nucleus and ventrally to the cerebral aqueduct, the common oculomotor nuclear complex (asterisk) can be appreciated.

At the caudal midbrain level (Fig. 2), the oval-shaped formation corresponding to the nigrosome 1 (3) is clearly distinguishable from the other portions of the substantia nigra because of its lower iron content. At this level, crus cerebri, medial lemniscus (oblique open black arrow), spinothalamic tract (horizontal open black arrow*)*, lateral lemniscus (horizontal white/black arrow) and central tegmental tracts (9) are also visible. The superior cerebellar peduncle (5) takes the place of the red nucleus at the level of the tegmentum.

At the level of the rostral pons (Fig. 3), the transverse pontocerebellar fibres (3) and the superior cerebellar peduncles (11) are clearly visible in both *ex vivo* and *in vivo* images. The lateral lemniscus (6/horizontal black arrow) is also visible and, together with the medial lemniscus (5) and the medial longitudinal fasciculus (black arrowhead), can be followed up to the midbrain. In the pontine tegmentum, the parabrachial nuclei (oblique black arrow and asterisk*)*, reticular nuclei of the pons (4, 7, 8) and the locus coeruleus (star) can be identified.

At the level of the caudal pons (Fig. 4), the abducens nucleus (9), fibres of the abducens nerve (arrowheads) and the intratruncal course of the facial nerve (oblique white/black arrow), forming the facial colliculus (vertical white/grey arrow*)* of the medial eminence, are detectable in the dorsal part of the pons also in *in vivo* images.

At the level of the rostral medulla oblongata (Fig. 5), the corticospinal tract (1) is detectable ventrally forming the medullary pyramids, the inferior olivary nucleus (4) anterolaterally and the spinal trigeminal nucleus (8) and the corresponding tract (9) are visible dorsolaterally. At the level of the medullary tegmentum, the hypoglossal nucleus (11) and the dorsally located dorsal longitudinal fasciculus (oblique black arrow) can be identified close to the midline, forming the hypoglossal trigone; laterally, the vagal trigone is formed by the dorsal motor nucleus of the vagus (12) and the dorsal nuclei of the solitary tract complex, while the solitary tract (oblique white arrow) can be identified more ventrally; more laterally, the vestibular nuclei (13) can be identified at the level of the acoustic and vestibular trigone of the medulla.

At the level of the caudal medulla oblongata (Fig. 6), right above the pyramidal decussation, gracile (10), cuneate (12) and spinal trigeminal nuclei (4) and the corresponding tracts (11, 13, 5) are clearly visible *in vivo*, especially using the χ map. Spinothalamic, ventral spinocerebellar and dorsal spinocerebellar tracts (3) are also recognisable but cannot be distinguished from each other. Particularly striking is the course of the internal arcuate fibres (8) originating from the gracile and cuneate nuclei, decussating posteriorly to the pyramids and forming the medial lemniscus (7).

**Fig. 7:**
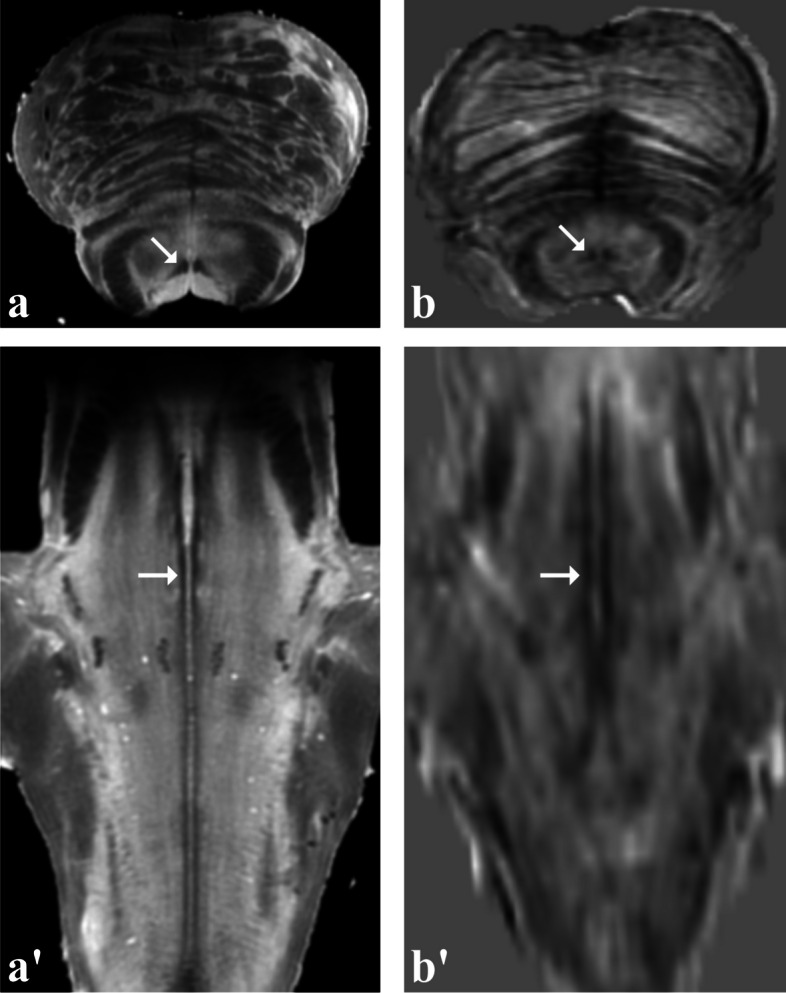
Images of the medial longitudinal fasciculus. Axial (**a**, **b**) and oblique coronal (**a**, **b’**) views of the medial longitudinal fasciculus (arrows) in the *ex vivo* sample (**a**, **a’**) and in the volunteer (**b**, **b’**)

Across axial, sagittal and coronal views, a number of fibre tracts were visible for most of their course, such as the medial lemniscus, the spinothalamic tract and the medial longitudinal fasciculus (Fig. 7).

For each of the selected anatomical levels, 16 to 21 anatomical structures were visible in *ex vivo* MR imaging, with a total of 104 structures, all of them confirmed with histological staining. Twenty-nine to 75% of them were visible also in *in vivo* T2*-weighted imaging and 63 to 94% in *in vivo* χ and frequency maps (Table 1). Overall, in *in vivo* acquisitions, advanced imaging (χ and frequency maps) performed better than T2*-weighted imaging and allowed visualising a greater number of anatomical structures, especially in the pons and medulla oblongata. All the brainstem substructures identified *in vivo* are confirmed with the *ex vivo* imaging and histological evaluation.

**Table 1:** Summary of the brainstem white matter tracts and grey matter nuclei visible at each selected anatomical level in both *ex vivo* and *in vivo* imaging

Anatomical level	*Ex vivo* imaging	*In vivo* T2*-weighted imaging	*In vivo* * χ*/frequency maps
Rostral midbrain (Fig. 1)	16	12 (75%)	10 (63%)
Caudal midbrain (Fig. 2)	16	11 (69%)	15 (94%)
Rostral pons (Fig. 3)	17	5 (29%)	13 (76%)
Caudal pons (Fig. 4)	21	6 (29%)	18 (86%)
Rostral medulla oblongata (Fig. 5)	20	7 (35%)	16 (80%)
Caudal medulla oblongata (Fig. 6)	14	7 (50%)	10 (71%)

When anatomical structures were visible but not distinguishable from each other (*e.g.*, the white matter tracts which constitute the cerebral peduncle), they were considered together as a single entity. Numbers in brackets indicate the percentage of anatomical structures visible in *ex vivo* imaging which is visible also in *in vivo* imaging

Using information derived by different types of multi-echo GRE images, it is possible to delineate the main components of the most important anatomo-functional networks of the brainstem.

**Fig. 8:**
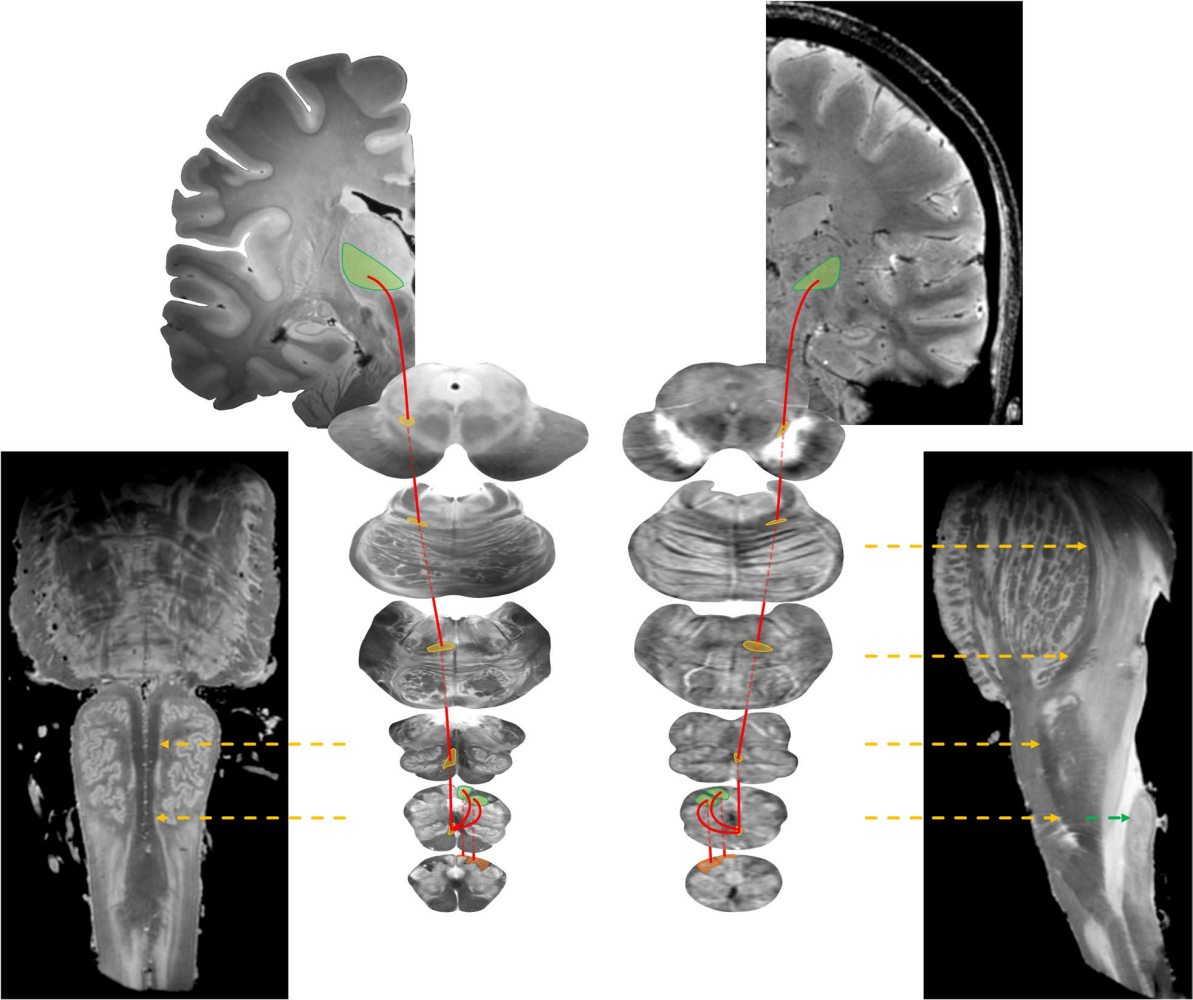
Representation of the dorsal column-medial lemniscus system. This sensory pathway (red lines) is represented in axial *ex vivo* (left) and *in vivo* (right) images from the caudal medulla oblongata to the midbrain and in the coronal view of the ventral posterior nucleus of thalamus. The medial lemniscus is highlighted in yellow across the axial images and pointed at by yellow arrows in the oblique sagittal and oblique coronal representative views of the brainstem; gracile nucleus, cuneate nucleus and ventral posterolateral nucleus of thalamus are highlighted in green; gracile and cuneate fasciculi are highlighted in orange

The dorsal column-medial lemniscus system is the sensory pathway conveying fine touch sensation (epicritic), vibratory sensation and conscious proprioception from the body. Along the brainstem, the first-order sensory neurons send their afferent fibres through the gracile and cuneate tracts (also known as the tracts of Goll and Burdach, respectively) in the medulla oblongata. The synapses with the second-order sensory neurons occur in the nucleus gracilis and cuneatus; then, the axons of the second-order neurons cross over the midline (sensory decussation, internal arcuate fibres), form the medial lemniscus and project to the ventral posterolateral nucleus of the thalamus (Fig. 8).

The sensitive anterolateral system is the other sensory pathway and conveys crude touch (protopathic), pain and temperature sensations from the body. The second-order sensory neurons cross the midline at the spinal level, form the spinothalamic tract and project to the ventral posterolateral nucleus of the thalamus.

The pyramidal tract is the main motor pathway controlling the voluntary movements of the body. It is made by the axons of upper motor neurons divided into two tracts, namely the corticospinal tract (CST), which conveys information to the trunk and limbs, and the corticonuclear tract, which projects to the brainstem nuclei and controls movements of the orofacial region. The axons of the upper motor neurons originate from the primary motor cortex and descend to the brainstem through the cerebral peduncles. At the level of the cerebral peduncles, the pyramidal tract cannot be distinguished from either the frontopontine tract of Arnold, placed medially or the parieto-temporo-occipito-pontine tract of Türck, laterally. In the rostral pons, the CST crosses the pontocerebellar fibres and is not clearly defined because it is split into small fascicles and embedded among the pontine nuclei. In the caudal pons and medulla oblongata, instead, CST fibres are bundled, and the shape of the tract is more defined, forming the medullary pyramids.

The dentato-rubro-olivary tract is a bidirectional extrapyramidal pathway which constitutes the triangle of Mollaret and, together with the CST, is involved in the regulation of automatic movements [26]. It is formed by dentate efferent fibres which ascend along the superior cerebellar peduncle to reach the rostral part (parvicellular) of the contralateral red nucleus. The efferent fibres of the red nucleus descend ipsilaterally via the central tegmental tract and reach the inferior olivary nucleus. Ascending decussating fibres originating from the inferior olivary nucleus complete the triangle, passing through the inferior cerebellar peduncle and terminating in the dentate nucleus.

## Discussion

T2*-weighted magnitude images, *χ* and frequency maps derived from a multi-echo GRE sequence at 7 T revealed brainstem structures usually evaluated through light microscopy, such as the accessory medial and dorsal olivary nuclei, oculomotor nucleus, solitary tract, medial and dorsal longitudinal fasciculus and the mesencephalic tract of the trigeminal nerve. Overall, images depicted the morphological organisation of the brainstem, as revealed by comparison with the histological assessment.

Magnetic susceptibility-based contrast in the brain originates primarily from paramagnetic (*e.g.*, iron) and diamagnetic (*e.g.*, myelin) substances, which have opposite susceptibility behaviour [27]. The sensitivity to susceptibility phenomena increases with increasing magnetic field strength, and small differences of susceptibility between grey and white matter brainstem substructures are enhanced at 7 T. Recently, T2*-weighted magnitude images [28], and even more χ maps [29], were proved to improve the *in vivo* detection of inner structures of the brainstem. In our study, the combined evaluation of T2*-weighted magnitude images, χ, and frequency maps derived from a 7-T MR acquisition allows delineating *in vivo* the small components of the main anatomic pathways in the brainstem.

The susceptibility-weighted imaging confirms its role in improving the contrast among midbrain structures compared to conventional sequences [12]. Indeed, it allows revealing the trilaminar organisation of the substantia nigra and the dorsolateral ovoid-shaped hyperintensity corresponding to nigrosome 1 formation [30, 31], differentiating between poor and densely vascularised components of the red nucleus [32] and identifying the subthalamic nucleus [33] as a structure distinct from the substantia nigra. On the other hand, frontopontine and temporo-pontine fibres cannot be separated from each other within the cerebral peduncles either in *in vivo* or *ex vivo* imaging [34].

The capability of χ and frequency maps in revealing grey and white matter substructures of the brainstem and distinguishing them from each other depends on several factors [9] such as deoxyhaemoglobin, noniron metals, fibres direction [35] and, mainly, the amount of iron and myelin content that have respectively paramagnetic and diamagnetic properties [27]. Therefore, differences in iron and myelin content among adjacent brainstem structures allow them to be identified as specific entities, but this capability is still suboptimal. Susceptibility-weighted imaging clearly depicts iron-rich grey matter nuclei, such as substantia nigra and red nuclei, but is sensitive also to nuclei with lower iron content, such as the spinal trigeminal nucleus and the inferior olivary nucleus. On the other hand, iron-poor nuclei [36] are probably visible with χ maps because they contain less myelin (and thus are less diamagnetic) than the surrounding white matter.

The direct visualisation of the anatomo-functional systems of the brainstem might facilitate the diagnostic process when signal alterations colocalise with specific anatomical structure. An example is the vitamin B12 deficiency, which is radiologically characterised by bilateral T2 hyperintensity in the location of the fasciculus gracile and cuneatus. The visualisation of these white matter tracts and their nuclei, and the detection of a signal alteration involving selectively these structures, might make the radiologist more confident in diagnosing the disease [37].

The description of the dentato-rubro-olivary system is a prerogative of microscopy [25]. So far, it has been partially identified *in vivo* using diffusion tensor imaging [38] and indirectly with conventional MR sequences if it is affected by degeneration [39]. Whereas red nuclei and dentate nuclei are visible with iron-sensitive techniques also at high magnetic fields, the use of a 7-T system make the direct and clear visualisation of the inferior olivary nuclei feasible, thus completing the study of the triangle of Guillain-Mollaret. This might be useful in patients with pathologies affecting this complex system or manifesting with symptoms pertaining to hypertrophic olivary degeneration such as palatal myoclonus [40].

From a therapeutical point of view, the possibility to better define margins of the subthalamic nucleus and parcellate its inner structure based on the iron content can be exploited to improve the direct targeting for deep brain stimulation [41]. Furthermore, the surgical trajectories for tumours excision can be planned more accurately if the topography of the inner substructures of the brainstem is known [42].

Although χ and frequency maps allow identifying a large number of brainstem substructures, *in vivo* imaging has not yet reached the level of detail achieved in *ex vivo* MR acquisitions for a number of reasons. Firstly, motion (respiration, pulsatility of arteries and cerebrospinal fluid) and susceptibility artifacts affect MR imaging at ultrahigh field, especially in the posterior fossa and basicranium, and hamper the study of the brainstem. Secondly, the spatial resolution we reached in the *ex vivo* sample with a customised small-bore coil is extremely challenging *in vivo* because of signal-to-noise and acquisition time constraints. Thirdly, *in vivo* relaxation times are different from those of *ex vivo* fixed samples [43, 44], and, therefore, the contrast between brainstem substructures might be suboptimal.

Last points to take into consideration in the *in vivo-ex vivo* comparison are the possible asymmetry of imaged structures, because of the difficulty to obtain a perfect orientation of homologous regions *in vivo*, and the small sizes and the low intrinsic contrast (low iron content of the nuclei and white matter tracts) of many structures in the caudal brainstem, which hamper the ability to differentiate them from each other. However, *χ* and frequency maps outperform T2*-weighted magnitude images in distinguishing small white matter tracts from grey matter nuclei, thus improving the differentiation between them also at level of medulla oblongata.

Post-mortem conventional MRI at high [45] and ultrahigh field [46, 47] has proved to be an extraordinary method for studying the brainstem anatomy, comparable to histological staining but with the added value of multiplanar reconstructions that improves learning brainstem anatomy for clinicians and trainees. Post-mortem MRI studies together with histological staining constitute an anatomical ground truth for the human brain and are fundamental for the comparison with *in vivo* neuroimaging [48].

Our study also suggests that in autoptical cases with small brainstem lesions, preliminary MRI could be very useful in identification and anatomical localisation of the lesions and in guiding brainstem sampling for following histopathological analysis. In this sense, it has been stressed also in Forensic Clinical Anatomy the importance of post-mortem imaging of single organs for implementation of anatomical definition of histopathological findings [49, 50].

To the best of our knowledge, this is the first study in which *in vivo* 7-T MR imaging of the brainstem was compared with both MR images and histological data of an *ex vivo* sample. All the brainstem substructures recognised in the *ex vivo* MRI were confirmed in the histological sections based on topography and morphology. Moreover, a correspondence between MR signal intensity of many brainstem substructures and their myelin density or iron load could be established. As mentioned before, the ability to distinguish between grey matter nuclei and white matter tracts with MRI relies mainly on their different content in myelin and iron. It is noteworthy that, overall, the greyscale contrast of histological sections stained with Weigert-Pal technique is similar to the one of PD-weighted images and frequency maps and opposite to the one of *χ* maps. Indeed, from one hand, myelin-rich structures (dark in sections stained with Weigert-Pal technique) have low χ and high-frequency values; on the other hand, grey and white matter have different number of water protons and, therefore, different signal intensity in PD-weighted images, with grey matter structures being hyperintense (*e.g.*, central grey matter, dorsal motor nucleus of the vagus, nucleus of the solitary tract) and white matter structures mildly to markedly hypointense (*e.g.*, corticospinal tract, superior cerebellar pedicles, cuneate fasciculus).

Although *in vivo* imaging does not capture all the substructures visible in *ex vivo* acquisitions, the considerable number of white matter tracts and grey matter nuclei which can be detected represents new and valuable landmarks for studying structures which are not visible with the current techniques, in addition to or in the place of the canonical ones, such as the anatomical levels of section and surface markers.

In conclusion, our pilot study at ultrahigh magnetic field evaluated the ability of T2*-weighted magnitude images, *χ* and frequency maps to resolve *in vivo* the anatomy of brainstem structures which are usually undistinguishable from each other. The capability of the proposed approach to detect the pathological involvement of specific anatomo-functional systems of the brainstem depends on the sensitivity of the acquisition protocol in depicting the brainstem anatomy and revealing signal changes. Further studies are needed to test and refine this type of imaging in pathological conditions.

### Supplementary Information

**Additional file 1: Supplementary Table S1. **Anatomical structures visualised in *in vivo* MR images and figures in which each of them is visible.

## Data Availability

All data generated or analysed during this study are included in this published article and its supplementary information file.

## Abbreviations

2D: Two-dimensional
3D: Three-dimensional
CST: Corticospinal tract
GRE: Gradient-recalled echo
MRI: Magnetic resonance imaging
NEX: Number of excitations
PD: Proton density
SE: Spin echo
TE: Time of echo
TR: Time of repetition
*χ*: Magnetic susceptibility
