# Supplementary material for: Brainstem anatomy with 7-T MRI: in vivo assessment and ex vivo comparison
Source: Eur Radiol Exp. 2023 Nov 16;7:71. doi: 10.1186/s41747-023-00389-y (PMC10651583; doi:10.1186/s41747-023-00389-y)
Supplement: Supplementary file 1 — Additional file 1: Supplementary Table S1. Anatomical structures visualised in in vivo MR images and figures in which each of them is visible. [file 41747_2023_389_MOESM1_ESM.docx]

**Brainstem anatomy with 7-T MRI: in vivo assessment and ex vivo comparison**

**ELECTRONIC SUPPLEMENTARY MATERIAL**

**Supplementary Table S1.** Anatomical structures visualised in *in vivo* MR images and figures in which each of them is visible

| **Anatomical structures** | **Figures in which each anatomical structure is visible** |
| --- | --- |
| corticospinal, corticonuclear, frontopontine and parietotemporopontine tracts | 1, 2 |
| pars reticulata of the substantia nigra | 1 |
| extension of the pars reticulata into the crus cerebri | 1 |
| subthalamic nucleus | 1 |
| ventral tegmental area | 1, 2 |
| magnocellular portion of the red nucleus | 1 |
| lamina medullaris of the red nucleus | 1 |
| parvocellular portion of the red nucleus | 1 |
| oculomotor, principal oculomotor and accessory oculomotor nuclei | 1 |
| cerebral aqueduct | 1 and 2 |
| periaqueductal grey matter | 1 and 2 |
| superior colliculus | 1 |
| substantia nigra | 2 |
| nigrosome 1 | 2 |
| superior cerebellar peduncle | 2 and 3 |
| medial lemniscus | 2, 4, 5 and 6 |
| spinothalamic tract | 2 |
| lateral lemniscus | 2 and 3 |
| nucleus reticularis cuneiformis and nucleus reticularis peduncolopontinus | 2 |
| central tegmental tract | 2 and 3 |
| tectospinal tract and medial longitudinal fasciculus | 2 |
| dorsal nucleus of raphe | 2 |
| inferior colliculus | 2 |
| pontine nuclei | 3 and 4 |
| pontocerebellar fibres | 3 and 4 |
| medial lemniscus and spinothalamic tract | 3 |
| nucleus reticularis centralis superior | 3 |
| nucleus reticularis pontis oralis | 3 |
| medial longitudinal fasciculus | 3, 4 and 5 |
| central grey matter | 3 |
| nucleus coeruleus | 3 |
| medial parabrachial nucleus and nucleus subcoeruleus | 3 |
| lateral parabrachial nucleus | 3 |
| corticospinal tract split into small fasciculi and corticonuclear tract | 4 |
| pontine reticularis nuclei | 4 |
| tectospinal tract | 4 and 5 |
| abducens nucleus | 4 |
| fibres of the abducens nerve | 4 |
| facial nucleus | 4 |
| fibres of the facial nerve | 4 |
| facial colliculus | 4 |
| medial and superior vestibular nuclei | 4 |
| lateral vestibular nucleus | 4 |
| spinal trigeminal nucleus | 4, 5 and 6 |
| spinal trigeminal tract | 4, 5 and 6 |
| middle cerebellar peduncle | 4 |
| inferior cerebellar peduncle | 4 |
| corticospinal tract | 5 |
| inferior olivary nucleus | 5 |
| amiculum of the inferior olivary nucleus | 5 |
| medial accessory olivary nucleus | 5 |
| dorsal accessory olivary nucleus | 5 |
| nucleus reticularis centralis medullae oblongatae | 5 and 6 |
| dorsal motor nucleus of the vagus and nucleus of the solitary tract | 5 |
| hypoglossal nucleus | 5 |
| medial vestibular nucleus | 5 |
| lateral cuneate nucleus | 5 |
| spinothalamic tract, ventral spinocerebellar tract, inferior cerebellar peduncle | 5 |
| spinothalamic tract, ventral spinocerebellar tract, dorsal spinocerebellar tract and rubrospinal tract | 6 |
| Internal arcuate fibres | 6 |
| central canal | 6 |
| nucleus of the solitary tract | 6 |
| gracile nucleus | 6 |
| gracile fasciculus | 6 |
| medial cuneate nucleus | 6 |
| cuneate fasciculus | 6 |
